# Neonatal circumcision availability in the United States: a physician survey

**DOI:** 10.1186/s12894-021-00911-7

**Published:** 2021-10-27

**Authors:** Ushasi Naha, Hans C. Arora, Ryan F. Walton, Ilina Rosoklija, Lindsay M. Skibley, Emilie K. Johnson

**Affiliations:** 1 University of Illinois College of Medicine, Chicago, USA; 2 Division of Urology, Ann & Robert H. Lurie Children’s Hospital of Chicago, Chicago, USA; 3 Department of Urology, Northwestern University Feinberg School of Medicine, 225 E Chicago Avenue, Box 24, Chicago, IL 60611 USA; 4 Department of Pediatrics, Northwestern University Feinberg School of Medicine, Chicago, USA; 5 Center for Health Services and Outcomes Research, Northwestern University Feinberg School of Medicine, Chicago, USA

**Keywords:** Neonatal, Circumcision, Access, Pediatrics, Pediatric urology

## Abstract

**Background:**

A significant proportion of boys present to surgical specialists later in infancy/early childhood for elective operative circumcision despite the higher procedural risks. This study aims to assess physician perspectives on access to neonatal circumcision across the United States and identify potential reasons contributing to disparities in access.

**Methods:**

A cross-sectional survey was electronically distributed to physician members of the Societies for Pediatric Urology and the American Academy of Pediatrics Section on Hospital Medicine. Hospital characteristics and circumcision practices were assessed. Associations between NC availability and institutional characteristics were evaluated using chi-squared testing and multivariable logistic regression. Qualitative analyses of free-text comments were performed.

**Results:**

A total of 367 physicians responded (129 urologists [41%], 188 pediatric hospitalists [59%]). Neonatal circumcision was available at 86% of hospitals represented. On univariate and multivariate analysis, the 50 hospitals that did not offer neonatal circumcision were more likely to be located in the Western region (odds ratio [OR] = 8.33; 95% confidence interval [CI] 3.1–25 vs. Midwest) and in an urban area (OR = 4.2; 95% CI 1.6–10 vs. suburban/rural) compared with hospitals that offered neonatal circumcision. Most common reasons for lack of availability included not a birth hospital (N = 22, 47%), lack of insurance coverage (N = 8, 17%), and low insurance reimbursement (N = 7, 15%). Institutional, regional, or provider availability (68%), insurance/payment (12.4%), and ethics (12.4%) were common themes in the qualitative comments.

**Conclusions:**

Overall availability of neonatal circumcision varied based on hospital characteristics, including geography. Information from this survey will inform development of interventions designed to offer neonatal circumcision equitably and comprehensively.

**Supplementary Information:**

The online version contains supplementary material available at 10.1186/s12894-021-00911-7.

## Introduction

Circumcisions are the most commonly performed pediatric surgical procedure [1]. For families who seek circumcision for their sons, health benefits include the decreased risk of urinary tract infections, sexually transmitted infections and penile cancer. These benefits are greatest for boys when the circumcision is performed in the neonatal period [2]. When circumcisions are done beyond the neonatal period, there are higher complication rates [3], and general anesthesia is required, leading to increased risks [4–8] and costs [9, 10].

Over time, neonatal circumcision rates in the United States (US) have modestly declined overall. However, boys with public insurance have had lower rates of circumcision compared to those with private insurance, even when controlling for demographics, geographic region, hospital and year of birth. Additionally, boys from lower-income families have lower rates of circumcision in comparison to boys from families with higher income [11]. This suggests that there are barriers in access underlying the differing rates of neonatal circumcision. Indeed, a study of free-standing children’s hospitals found that 28% of boys undergoing delayed surgical circumcisions were Black/African–American, and 58% of boys had public insurance [12]. After the defunding of Medicaid coverage for circumcision, state-specific rates of neonatal circumcision decreased by around 20%. In states and years without Medicaid coverage, Black infants had lower odds of undergoing neonatal circumcision [13].

Though prior data indicate differences in neonatal circumcision access by demographic factors including income, insurance type, and race, the precise reasons for these differences have not been identified. Differences in availability and efficiency of neonatal circumcision at birth hospitals may be one factor contributing to the apparent disparity in access to neonatal circumcision. The present study aims to assess physician perspectives on access to neonatal circumcision at hospitals throughout the US, and identify potential underlying reasons contributing to disparities in access to the procedure. Our hypothesis is that neonatal circumcision availability will differ based on the geographic location and by hospital characteristics.

## Methods

### Overview

A survey study of physicians who practice at hospitals that care for newborns was conducted. The primary outcome was neonatal circumcision availability by hospital. Survey content also aimed to describe features of hospitals that perform newborn circumcision, including those that could contribute to limited circumcision availability at certain times, or for certain patients.

### Survey development

A 21-question survey was developed to ascertain hospital characteristics and circumcision practices of institutions that care for newborns across the US (Additional file 1). Hospital characteristics assessed included the teaching and metropolitan status, geographic region and whether the institution was public or private. We also assessed whether hospitals had a standard circumcision protocol used during the birth encounter. Availability of circumcision, including details of time, dates, payment options and specialties that perform circumcisions, as well as circumcision exclusion criteria, were collected. The last question was a free-text question where respondents could add any further comments about the availability of neonatal circumcisions at their institution or in their region. The study was approved by the Ann & Robert H. Lurie Children’s Hospital of Chicago Institutional Review Board and the need for obtaining informed consent from subjects was waived (IRB 2020-3505).

### Survey distribution

A cross-sectional, anonymous survey was electronically distributed to physician members of the Societies for Pediatric Urology (SPU) and the American Academy of Pediatrics (AAP) Section on Hospital Medicine through organizational email lists. Responses were collected starting on March 31 until May 12, 2020. One reminder email was sent to AAP members and two reminder emails were sent to the SPU email lists. Survey responses were recorded and stored without participant identifiers using REDcap [14].

### Analysis

Descriptive statistics were used to summarize survey respondents and characteristics at institutions that perform circumcisions. Univariate comparisons of hospital characteristics and circumcision practices between institutions that do and do not perform circumcisions were performed using Pearson chi-square tests. Multivariable logistic regression was performed to assess adjusted associations between institutional characteristics and circumcision availability. Covariates were initially determined a priori, taking into account the statistical significance of covariates in the univariate analysis. Covariates in the regression model included geographic region, private versus public institution, metropolitan status and teaching status. Regression analyses were displayed using forest plots. All statistical analyses were performed using R 4.0.0 (R Foundation for Statistical Computing, Vienna, Austria) using 95% confidence intervals with two-sided *p* values < 0.05 considered significant. An inductive, qualitative analysis of free-text comments about neonatal circumcision availability was performed, and key themes were summarized.

## Results

### Cohort characteristics

A total of 367 physicians (165 pediatric urologists [45%], 202 pediatric hospitalists [55%]; Table 1) responded to the survey, with an estimated response rate of 9.5% (pediatric hospitalists: 6%; pediatric urologists: 23%). Response rates were estimated based on the number of clinicians on the organizational email lists. All completed surveys were included in the analysis. Most respondents (317/367 [86%]) reported neonatal circumcision availability at their institution. The geographic distribution by US region was relatively even (South: 31%, Midwest: 28%, West: 23%, Northeast: 17%). Most respondents worked at private, non-profit institutions (66%) in urban settings (66%) that were teaching hospitals (82%).

**Table 1:** Univariate analysis of respondents and hospital characteristics

	Total (N = 367)	Circumcisions offered (N = 317)	Circumcisions not offered (N = 50)	*p* value
Region				< 0.001*
Midwest	101 (27.5%)	95 (30.0%)	6 (12.0%)	
Northeast	62 (16.9%)	61 (19.2%)	1 (2.0%)	
South	114 (31.1%)	102 (32.2%)	12 (24.0%)	
West	85 (23.2%)	58 (18.3%)	27 (54.0%)	
Other	4 (1.1%)	1 (0.3%)	3 (6.0%)	
No response	1 (0.3%)	0 (0.0%)	1 (2.0%)	
Type of institution				0.307
Private for profit	36 (9.8%)	29 (9.1%)	7 (14.0%)	
Private non-profit	243 (66.2%)	206 (65.0%)	37 (74.0%)	
Public	69 (18.8%)	63 (19.9%)	6 (12.0%)	
Uncertain	14 (3.8%)	14 (4.4%)	0 (0.0%)	
Other	4 (1.1%)	4 (1.3%)	0 (0.0%)	
No response	1 (0.3 %)	1 (0.3%)	0 (0.0%)	
Metropolitan status				0.002*
Suburban/rural	124 (33.8%)	118 (37.2%)	6 (12.0%)	
Urban	241 (65.7%)	197 (62.1%)	44 (88.0%)	
Other	2 (0.5%)	2 (0.6%)	0 (0.0%)	
Teaching status				0.885
Non-teaching	59 (16.1%)	50 (15.8%)	9 (18.0%)	
Teaching	301 (82.0%)	261 (82.3%)	40 (80.0%)	
Don’t know/uncertain	5 (1.4%)	4 (1.3%)	1 (2.0%)	
No response	2 (0.5%)	2 (0.6%)	0 (0.0%)	
Respondent specialty				< 0.001*
Pediatric urology	165 (45.0%)	129 (40.7%)	36 (72.0%)	
Pediatric hospitalist	202 (55.0%)	188 (59.3%)	14 (28.0%)	

*Indicates statistical significance

### Comparison of institutions by circumcision availability

**Fig. 1:**
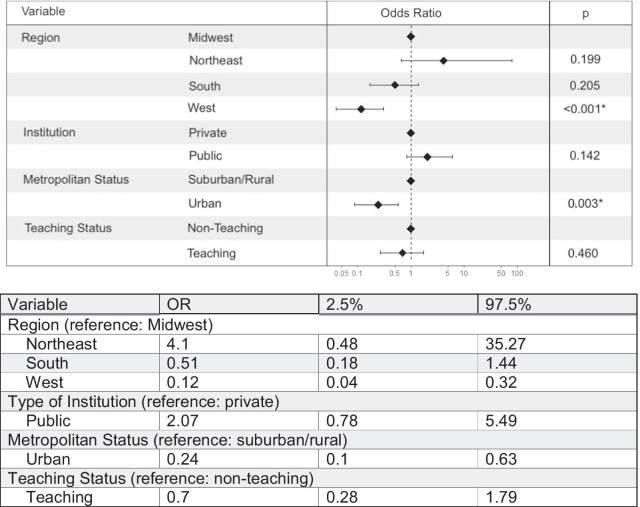
Factors associated with institutions that offer circumcisions

Table 1 compares characteristics of respondents and hospitals offering versus not offering neonatal circumcisions. More pediatric hospitalists than pediatric urologists were from institutions that offered circumcisions (59% vs. 41%), whereas 72% of respondents from institutions that did not offer circumcisions were pediatric urologists (vs. 28% pediatric hospitalists; *p* < 0.001). A difference by geographic region was also observed: hospitals in the South were most frequently represented among hospitals that offered circumcisions whereas hospitals in the Western region were most represented among hospitals that did not offer circumcisions (*p* < 0.001). Urban institutions were more highly represented among hospitals that did not offer circumcisions compared to those that do (88% vs. 62%, respectively, *p* = 0.002). On multivariate analysis, the 50 hospitals that did not offer neonatal circumcision were more likely to be located in the Western region (odds ratio [OR] = 8.33; 95% confidence interval [CI] 3.1–25 vs. Midwest), and in an urban area (OR = 4.2; 95 % CI 1.6–10 vs. suburban/rural), compared with hospitals that offered the procedure (Fig. 1).

### Institutional factors

**Fig. 2:**
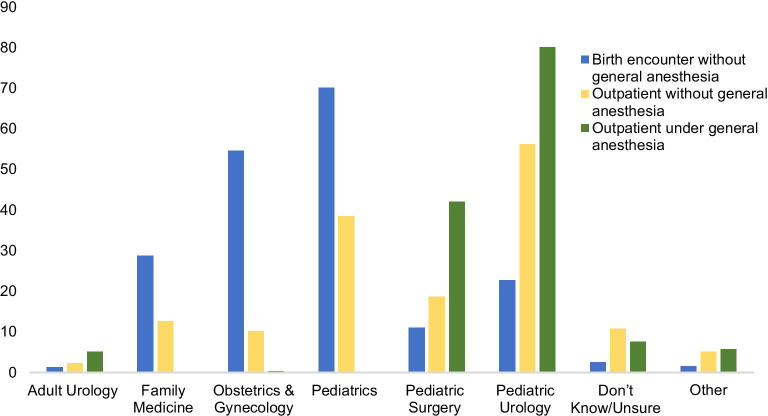
Specialties that perform circumcisions (N = 317)

Table 2 displays characteristics of hospitals that offer neonatal circumcision. A standardized protocol/checklist was available at less than half of hospitals. Many specialties performed circumcisions including Pediatrics (74%), Obstetrics and Gynecology (57%), Pediatric Urology (54%), Family Medicine (31%) and Pediatric Surgery (31%), with variability depending on setting and type of anesthesia (Fig. 2). Pediatric Urology most frequently performed outpatient procedures without or under general anesthesia (56% and 80%, respectively), while Pediatrics most commonly performed circumcisions at the birth encounter without general anesthesia (70%). Hospitals offering neonatal circumcision had more availability on weekdays versus weekends, and only 21% offered the procedure 24 h per day. Most hospitals reported accepting private insurance (79%) and slightly fewer accepted Medicaid (64%) for neonatal circumcision. Over half (56%) reported that at least some patients paid cash for the procedure.

**Table 2:** Characteristics at institutions that perform circumcisions (N = 317)

Characteristics	N (%)
Standard protocol, checklist, or similar that is used during the birth encounter that includes offering or performing circumcision	
Yes	139 (43.4%)
No	133 (41.6%)
Don’t know/unsure	44 (13.8%)
No response	1 (0.3%)
Who must initiate conversations about circumcision?	
Either hospital team or family	250 (78.9%)
Family	20 (6.3%)
Hospital team	10 (3.2%)
Don’t know/uncertain	6 (1.9%)
Other	31 (9.8%)
Entity that developed this protocol, checklist, or similar	
Institution	59 (18.4%)
Department	34 (10.6%)
Group practice	21 (6.6%)
Self	6 (1.9%)
Government	1 (0.3%)
Don’t know/unsure	106 (33.1%)
Other^a^	6 (1.9%)
No response	84 (26.3%)
Exclusion criteria at birth encounter for neonatal circumcision	
Penile anatomic abnormality (e.g., hypospadias, curvature, buried penis)	312 (97.5%)
Family history of bleeding disorder	233 (72.8%)
Prematurity	47 (14.7%)
Weight limit	38 (11.9%)
Older age at discharge	35 (10.9%)
Neonatal intensive care unit	25 (7.8%)
Other exclusion^b^	48 (15.0%)
Days that neonatal circumcision is available	
Monday	262 (82.6%)
Tuesday	261 (82.3%)
Wednesday	262 (82.6%)
Thursday	261 (82.3%)
Friday	261 (82.3%)
Saturday	246 (77.6%)
Sunday	242 (76.3%)
Don’t know/unsure	55 (17.4%)
Times neonatal circumcision is available during birth encounter	
Regular business hours (approximately 08:00 AM–05:00 PM)	165 (51.6%)
24 h per day	67 (20.9%)
Don’t know/unsure	56 (17.5%)
Other^c^	25 (7.8%)
No response	4 (1.3%)
Specialty that performs neonatal circumcisions at institution	
Pediatrics	233 (73.5%)
Obstetrics and gynecology	181 (57.1%)
Pediatric urology	170 (53.6%)
Family medicine	98 (30.9%)
Pediatric surgery	97 (30.6%)
Adult urology	9 (2.8%)
Other^d^	7 (2.2%)
Don’t know/unsure	3 (0.9%)
Payment for neonatal circumcision	
Private insurance	251 (79.4%)
Medicaid	205 (64.9%)
Self-pay/cash	177 (56.0%)
Don’t know/unsure	53 (16.8%)
Other^e^	6 (1.9%)

^a^Other entities: labor and delivery department, individual hospital unit, and pediatric urology

^b^Other exclusions: refusal of Vitamin K, heart murmurs, hyperbilirubinemia, oxygen monitoring and poor feeding

^c^Other time ranges: 8 am–9 am, 8 am–10 pm, every day at noon, only in the mornings and based on the availability of providers performing circumcisions,

^d^Other specialties: general surgery, hospitalist (adult, pediatric), midwife, neonatology, nurse practitioner

^e^Other payments: covered benefit through plan, hospital writes off the cost or covers if Medicaid, military, and Tricare

**Fig. 3:**
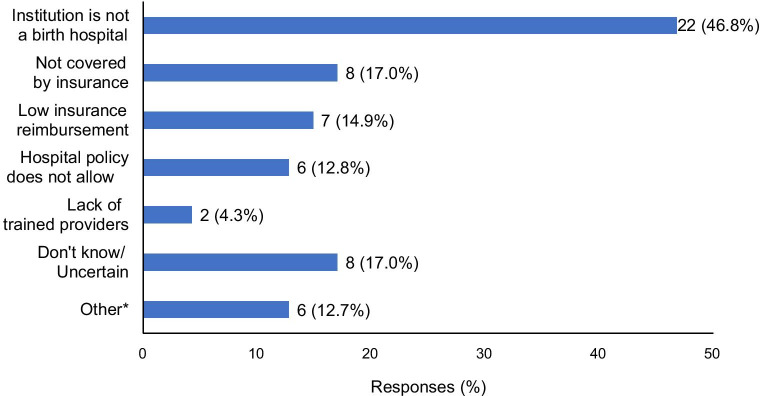
Reasons institutions do not offer neonatal circumcisions. *Other reasons included high inpatient volume (1), high facility charges (1) and because providers/specialties stopped offering (2)

For the 50 respondents from hospitals that did not offer neonatal circumcision, the most common reasons cited for lack of availability included: not a birth hospital (N = 22, 47%), lack of insurance coverage (N = 8, 17%), and low insurance reimbursement (N = 7, 15%), (Fig. 3).

### Qualitative comments

There were 105 free-text comments left by respondents (Table 3) when they were asked, “Do you have any other comments about availability of circumcision at your institution or in your region?”. Institutional, regional, or provider availability (N = 71, 68%), insurance and payment (N = 13, 12.4%), and ethics (N = 13, 12.4%) were the most common themes. Within the availability and payment themes, there were varied responses that highlighted either the ease or barriers to neonatal circumcision availability. Regarding ethics, several respondents indicated that they had ethical concerns with performing circumcisions as a routine/elective procedure.

**Table 3:** Themes of respondents’ free-text comments (N = 105)

Theme	Frequency	Representative auotation
Circumcision availability at institution or region	41 (39.0%)	*“If a family wants to have it done it is easy to get it performed”* *“There are no in-hospital postpartum circumcisions in the state of Nevada”*
Provider availability	30 (28.6%)	*“Providers can opt out of requested circumcision. This is increasing and parents are left to find circumcision services”* *“I get a lot of outpatient referrals because the rounding OB wasn’t ‘comfortable’ performing circs or logistically there wasn’t time to have it done before the family was discharged.”*
Insurance and payment	13 (12.4%)	*“The major limitation is financial - may families can not afford to self-pay, and Medicaid and most private insurances do not cover neonatal circumcisions in our state.”* *“…we accept Medicaid reimbursement so patients then don’t have to pay anything. We really [don’t] let it [be] known too much that we do [them] in the office and don’t charge Medicaid because it would be too many coming.”*
Ethics and personal opinions	13 (12.4%)	*“If this is being performed for cosmetic reasons only, it should not be permitted”* *“Just laying out my own bias that we should not do these (routine elective) procedures anymore. Sure there are indications and personal/cultural reasons but they could be outpatient.”*
Circumcision eligibility	11 (10.5%)	*“Excluded if family refuses Vitamin K at birth”* *“Some OBs will not do the circumcision, for prematurity (arbitrary cutoff), if the penis is too small, or if they are NICU.”*
Uncertainty about circumcision practices	6 (5.7%)	*“Unfortunately, not sure of exact weight limit at my institution”* *“Unsure what the practice is for premature infants at my institution”*

## Discussion

This survey of US pediatric urologists and hospitalists indicates that neonatal circumcision availability and practices vary by hospital, region, and specialty. Approximately 1 in 6 respondents were from hospitals that do not offer neonatal circumcision. Multiple specialties performed neonatal circumcisions; pediatric urologists most frequently performed outpatient procedures with or without general anesthesia, and pediatrics most frequently performed circumcisions at the birth encounter without general anesthesia. At institutions that did offer circumcision, availability was most common during the week and during regular business hours. Almost 1 in 3 respondents added free-text comments, indicating general interest in the topic of neonatal circumcision availability. The comments provided additional insight about circumcision availability in their institution or region, provider availability or payment.

Information from the current survey study confirms findings of existing large database investigations about circumcision practice patterns and trends. Regarding circumcision practitioner specialty, the present study findings are similar to a recent study using Pediatric Health Information Systems data by Many et al. which showed that pediatrics, obstetrics-gynecology, and perinatal medicine most commonly performed neonatal circumcisions. Beyond the neonatal period, pediatric urologists most commonly performed circumcisions in the study by Many [15], a finding also echoed in our survey. The regional and insurance coverage trends indicated by the present survey are also aligned with prior studies using national databases. While American Academy of Pediatrics recommends coverage for all desired neonatal circumcisions [2], a recent study by Navia et al. shows that male infants with private insurance had higher rates of neonatal circumcision [13]. Our study respondents indicate that private insurance is more frequently accepted as payment for neonatal circumcision, and that some families pay cash; thus financial barriers to desired circumcisions appear to exist for families with fewer financial resources.

The present study also adds the clinician perspective to data from prior parent surveys about newborn circumcision access and reasons for delay. In the present physician survey, the most common reasons neonatal circumcision was not offered included the institution was not a birth hospital, the procedure was not covered by insurance, and low insurance reimbursement. Similarly, Jacobson et al. found that among patients seeking elective circumcision, one of the most common reasons for delay in desired neonatal circumcision in the Chicago area was that the hospital or physician did not perform neonatal circumcision; additional factors included prematurity, penile abnormality and low birth weight [16]. A 2016 survey of parents in San Antonio seeking delayed circumcision for boys less than 2 years old reported reasons for delay of circumcision beyond the newborn period included impaired proceduralist availability and lack of circumcision availability at the birth hospital [17].

Our study results reinforce findings in the larger body of literature that specialty/institutional availability and payment coverage are crucial factors in determining access to neonatal circumcision. To gain a more comprehensive understanding of why certain institutions experience limited circumcision availability, we are currently conducting a qualitative interview-based study of clinicians who perform circumcisions about this topic. Future studies will also focus on the development of efficient neonatal circumcision workflows for different hospital types and determine the most optimal recommendations for public and private insurance coverage of neonatal circumcision.

While the current study provides an important physician perspective on the topic of access to neonatal circumcision, there are study limitations. The survey response was relatively low, particularly amongst pediatric hospitalists, which could limit generalizability of findings. However, findings largely align with previous studies about both circumcision trends and reasons for difficulty with access, suggesting the validity of the findings. Also, percentage of survey respondents by region relatively closely align to 2019 US Census data [18]. Respondents only included pediatric urologists and pediatricians, and therefore does not represent all specialties that perform circumcisions. For some survey questions, around 15–25 % of respondents were uncertain or did not respond. As such, insight about certain subtopics was suboptimal, including questions about a standardized protocol, exclusion criteria for neonatal circumcision, days of the week that circumcision were available, and reasons that institutions did not offer neonatal circumcision. Limited demographic information was ascertained for the sake of brevity and to maintain the anonymity of respondents. Therefore, some respondents could represent the same institutions, thus creating redundancy in responses.

## Conclusions

The availability of neonatal circumcision varies based on hospital characteristics, including geographic location. Specialties that perform neonatal circumcision depend on setting and type of anesthesia used. Follow up research is currently focused on the institutional level to better understand potential local barriers to neonatal circumcision availability. The eventual goal is the creation of hospital-based and financial incentive structural strategies to ensure equitable access for neonatal circumcisions.

## Supplementary Information

**Additional file 1.** Circumcision survey.

## Data Availability

The datasets generated and/or analyzed during the current study are available from the corresponding author on request.

## Abbreviations

AAP: American academy of pediatrics
CI: Confidence interval
OR: Odds ratio
SPU: Societies for pediatric urology
US: United States
