# Supplementary material for: Neonatal circumcision availability in the United States: a physician survey
Source: BMC Urol. 2021 Oct 27;21:148. doi: 10.1186/s12894-021-00911-7 (PMC8549161; doi:10.1186/s12894-021-00911-7)
Supplement: Supplementary file 1 — Additional file 1. Circumcision survey. [file 12894_2021_911_MOESM1_ESM.pdf]

Supplement 1 – Circumcision Survey

Survey Instructions

Thank you for agreeing to participate in this survey of access to newborn circumcision in the United States. The purpose of this survey is to assess institutional and regional practices related to circumcision in healthy infants who have no major medical problems. Questions should be answered with regards to typical, routine practices at the institution at which you practice primarily. If you practice at multiple institutions, please answer with respect to your primary affiliation or the institution at which you spend the greatest number of hours per month. For the purposes of this survey, please refer to the following definitions.

- "Birth encounter": the episode of inpatient care bounded from when the child is born to the time of discharge.
- "Neonatal circumcision": removal of the preputial skin of the penis performed by any technique under local anesthesia which occurs during the birth encounter.

Section 1: About You

1) In which state are you located? Please use two-letter postal code __ __

2) What is your specialty?
   - ☐ Pediatric Urology
   - ☐ Adult Urology
   - ☐ Pediatric Surgery
   - ☐ Obstetrics & Gynecology
   - ☐ Family Medicine
   - ☐ Pediatrics
   - ☐ Other (Please Specify): ____________________

3) Please indicate how many years ago you completed your training. Residency or Fellowship _____

Section 2: About Your Hospital

4) Is your institution public or private?
   - ☐ Public or government (e.g. county hospital)
   - ☐ Private, not-for-profit/non-profit
   - ☐ Private, for profit
   - ☐ Don't know/Unsure
   - ☐ Other (Please Specify): ____________________

5) Please indicate the metropolitan status of your institution:
   - ☐ Urban inner-city
   - ☐ Urban, not inner-city
   - ☐ Suburban
   - ☐ Rural
   - ☐ Other (Please Specify): ____________________

6) What is the teaching status of your institution?
   - ☐ Teaching
   - ☐ Non-teaching
   - ☐ Don't know/Unsure

Section 3: About Circumcision

7) Who must initiate the conversation about circumcision at your institution?

- ☐ Hospital team must initiate conversation
- ☐ Family or parent must initiate conversation
- ☐ Either hospital team or patient can initiate conversation
- ☐ Other (Please Specify): ____________________
- ☐ Don’t Know/Unsure

8) Are neonatal circumcisions performed at your institution during the birth encounter?
   - ☐ Yes – Continue to Question 9
   - ☐ No – Skip to Question 20
   - ☐ Don’t know/Unsure – Continue to Question 9

9) Is there a standard protocol, checklist, or similar that is used during the birth encounter that includes offering or performing circumcision?
   - ☐ Yes – Continue to Question 10
   - ☐ No – Skip to Question 11
   - ☐ Don’t know/Unsure – Continue to Question 10

10) If a standard protocol, checklist, or similar is used, by which entity is it produced?
   - ☐ Institution – Skip to Question 12
   - ☐ Department – Skip to Question 12
   - ☐ Group practice – Skip to Question 12
   - ☐ Government – Skip to Question 12
   - ☐ Self – Skip to Question 12
   - ☐ Other (Please Specify): ____________________ – Skip to Question 12
   - ☐ Don’t Know/Unsure – Skip to Question 12

11) At which visit are circumcisions available to be performed at your institution? Please check all that apply.
   - ☐ Birth encounter (without general anesthesia)
   - ☐ Outpatient visit under local anesthesia (typically within newborn period / within 6-8 weeks of age)
   - ☐ Outpatient visit under general anesthesia (typically after newborn period / older than 8 weeks of age)
   - ☐ Don’t Know/Unsure
   - ☐ Other (Please Specify): ____________________

12) Which specialty(ies) perform(s) neonatal circumcisions at your institution? Please check all that apply.
   - ☐ Pediatric Urology
   - ☐ Adult Urology
   - ☐ Pediatric Surgery
   - ☐ Obstetrics & Gynecology
   - ☐ Family Medicine
   - ☐ Pediatrics
   - ☐ Don’t Know/Unsure
   - ☐ Other (Please Specify): ____________________

13) At your institution, which specialty(ies) perform(s) circumcision during the <u>birth encounter (without general anesthesia)</u>? Please check all that apply.
   - ☐ Pediatric Urology
   - ☐ Adult Urology
   - ☐ Pediatric Surgery
   - ☐ Obstetrics & Gynecology
   - ☐ Family Medicine
   - ☐ Pediatrics
   - ☐ Don’t Know/Unsure
   - ☐ Other (Please Specify): ____________________

14) At your institution, which specialty(ies) perform(s) circumcision as an outpatient (without general anesthesia) (typically within the newborn period / within 6-8 weeks of age)? Please check all that apply.
- ☐ Pediatric Urology
- ☐ Adult Urology
- ☐ Pediatric Surgery
- ☐ Obstetrics & Gynecology
- ☐ Family Medicine
- ☐ Pediatrics
- ☐ Don't Know/Unsure
- ☐ Other (Please Specify): ____________________

15) At your institution, which specialty(ies) perform(s) circumcision as an outpatient under **GENERAL** anesthesia (typically after the newborn period / older than 8 weeks of age)? Please check all that apply.
- ☐ Pediatric Urology
- ☐ Adult Urology
- ☐ Pediatric Surgery
- ☐ Obstetrics & Gynecology
- ☐ Family Medicine
- ☐ Pediatrics
- ☐ Don't Know/Unsure
- ☐ Other (Please Specify): ____________________

16) How are patients able to pay for neonatal circumcision at your institution? Please check all that apply.
- ☐ Medicaid
- ☐ Private insurance
- ☐ Self-pay/cash
- ☐ Don't Know/Unsure
- ☐ Other (Please Specify): ____________________

17) If neonatal circumcision IS offered at your institution during the birth encounter, on what days is it available? Please check all that apply.
- ☐ Monday
- ☐ Tuesday
- ☐ Wednesday
- ☐ Thursday
- ☐ Friday
- ☐ Saturday
- ☐ Sunday
- ☐ Don't Know/Unsure

18) If neonatal circumcision IS offered at your institution during the birth encounter, at what times is it available?
- ☐ 24 hours per day
- ☐ Regular business hours (approximately 8:00 AM – 5:00 PM)
- ☐ Don't Know/Unsure
- ☐ Other (Please Specify): ____________________

19) If neonatal circumcision IS offered at your institution during the birth encounter, which of the following restrictions exclude a patient from eligibility? Please check all that apply.
- ☐ Penile anatomic abnormality (e.g., hypospadias, curvature, buried penis)
- ☐ Family history of bleeding disorder
- ☐ Prematurity
  - If **Prematurity**, then not eligible if born at or before ___weeks (Range: 23 - 37)
- ☐ Neonatal Intensive Care Unit stay
- ☐ Older age at discharge
  - If **Older age at discharge**, then not eligible if > ___weeks adjusted age (Range: 40 - 100)
- ☐ Weight limit

- If **Weight limit**, then not eligible if >____kg (Range: 3.0 - 10.0)

- ☐ Other exclusion (Please Specify): ____________________

20) If neonatal circumcision is NOT offered at your institution during the birth encounter, why not? Please check all that apply.
   - ☐ Hospital policy does not allow neonatal circumcision
   - ☐ Lack of trained circumcision providers
   - ☐ Neonatal circumcision not covered by insurance
   - ☐ Low insurance reimbursement
   - ☐ Institution is not a birth hospital/deliveries are not routinely performed here (e.g. children's hospital)
   - ☐ Don't Know/Unsure
   - ☐ Other (Please Specify): ____________________

21) Do you have any other comments about availability of circumcision at your institution or in your region?
